# Prolactin-related adverse events and change in prolactin levels in pediatric patients given antipsychotics for schizophrenia and schizophrenia spectrum disorders: A systematic review

**DOI:** 10.1186/s12887-016-0710-y

**Published:** 2016-11-09

**Authors:** Eric Druyts, Michael J. Zoratti, Kabirraaj Toor, Ping Wu, Salmaan Kanji, Kiran Rabheru, Edward J. Mills, Kristian Thorlund

**Affiliations:** 1 Faculty of Health Sciences, University of Ottawa, Ottawa, ON Canada; 2 Department of Clinical Epidemiology and Biostatistics, McMaster University, Hamilton, ON Canada; 3 Department of Pharmacy of the Ottawa Hospital, Ottawa Hospital Research Institute, Ottawa, ON Canada; 4 Department of Psychiatry, Ottawa Hospital, Ottawa, ON Canada

**Keywords:** Schizophrenia, Pediatric, Prolactin, Systematic review

## Abstract

**Background:**

Second-generation antipsychotics are commonly prescribed for pediatric patients with schizophrenia and schizophrenia spectrum disorders despite their lack of approval for use in children. Although considered a safer alternative to first-generation antipsychotics, there is evidence to suggest that second-generation antipsychotics may be associated with some adverse events as well as an increase in prolactin levels. The purpose of this review is to examine the risk of prolactin-related adverse events in pediatric patients using antipsychotics and to quantify changes in prolactin for this population.

**Methods:**

Literature searches were conducted in Medline, Embase, the Cochrane Central Register of Controlled Trials, and PsycINFO databases, supplemented with review of select gray literature to identify both randomized controlled trials and observational studies on pediatric patients prescribed antipsychotic medications for schizophrenia or schizophrenia spectrum disorders. Using a narrative approach, data on adverse events were recorded and changes from baseline in prolactin were pooled, where possible, from the randomized trials. Change from baseline in prolactin was evaluated for each treatment, as well as in comparison to placebo and to other treatments. Where data was available, these changes were evaluated separately for male and female patients.

**Results:**

Six randomized controlled trials and five observational studies, all examining the effects of second-generation antipsychotics, were selected. Literature reporting the effects of risperidone, quetiapine, aripiprazole, olanzapine, and paliperidone was identified, with varying doses. Prolactin-related adverse events were sparsely reported across studies. In evidence gathered from randomized controlled trials, risperidone, olanzapine, and two doses of paliperidone (3–5 mg/day and 6–12 mg/day) were associated with increased prolactin levels compared to baseline. With the exception of paliperidone, similar trends were observed in males and females, separately. The findings of the observational evidence served to both complement and run contrary to the randomized trials, with discrepancies attributed to differences in patient and treatment characteristics.

**Conclusions:**

No definitive conclusions between second-generation antipsychotic use and prolactin-related adverse events can be made based on the available literature. While some trends in prolactin level changes emerged, this was based on few trials with small sample sizes. Future investigations should emphasize reporting on treatment safety.

**Trial registration:**

PROSPERO CRD42014009506.

**Electronic supplementary material:**

The online version of this article (doi:10.1186/s12887-016-0710-y) contains supplementary material, which is available to authorized users.

## Background

First-generation antipsychotics (FGAs), developed in the 1950s, were the first class of antipsychotics to be prescribed for schizophrenia. However, FGAs were associated with adverse events such as extrapyramidal motor control disabilities [1]. Second-generation antipsychotics (SGAs) were developed with the aim of providing similar or greater efficacy in the reduction of schizophrenia-related symptoms with a reduction in adverse events.

Of the men and women diagnosed with schizophrenia, 40 % and 23 %, respectively, will be diagnosed before the age of 19 [2]. While SGAs have not been approved for use in the Canadian pediatric population [3], they are nevertheless widely prescribed for these patients [4, 5]. Between 1999 and 2008 antipsychotic use in Canada for patients aged 18 years or younger increased from 1.9 per 1000 to 7.4 per 1000 [6]. However, while SGAs are considered a safer alternative to FGAs due to the reduced tendency to induce adverse neurological effects, other equally problematic adverse events have been associated with SGAs, especially in the pediatric population [7].

Hyperprolactinemia may become a clinical concern when patients are prescribed medications that block the inhibition of prolactin secretion, such as through interference with tuberoinfundibular dopamine pathway [8]. In 2011, the Canadian Alliance for Monitoring Effectiveness and Safety of Antipsychotics in Children published a report on the association between elevated prolactin levels and adverse events such as gynecomastia, galactorrhea, menstrual irregularities, sexual dysfunction, and decreased libido [9]. The effect of prolactin on gynecomastia in men and galactorrhea in women has also been reported elsewhere [10].

The effect of antipsychotics to raise prolactin levels have been summarized in a review by Byerly et al. [11]. Risperidone, in particular, has been associated with a greater than normal elevation in prolactin levels compared to other SGAs in both pediatric and adult populations [11–15]. Despite lack of approval, there is considerable off-label use of risperidone in Canadian pediatric patients [16].

The primary objective of this review is to examine the risk of prolactin-related adverse events associated with the use of antipsychotic medications for the treatment of schizophrenia and schizophrenia spectrum disorders in pediatric patients. The secondary objective is to examine changes in prolactin levels associated with the use of antipsychotic medications in this patient population.

## Methods

### Search strategy

The systematic review protocol (PROSPERO CRD42014009506) has been described elsewhere [17]. Briefly, clinical literature databases were systematically searched to identify randomized controlled trials (RCTs) and observational studies evaluating changes in prolactin levels and prolactin-related adverse events in pediatric patients, aged 5 to 18 years, diagnosed with schizophrenia or schizophrenia spectrum disorders and treated with first- or second-generation antipsychotics. For studies that included a control group, eligible comparators included first- and second-generation antipsychotics or placebo. The databases searched include: Medline, Embase, Cochrane Central Register for Controlled Trials, PsycINFO, ClinicalTrials.gov, the International Clinical Trials Registry Platform, and the Drug Industry Document Archive.

### Study selection

Two reviewers, working independently, scanned all abstracts and relevant material identified in the literature search. The same two reviewers independently reviewed relevant abstracts in full-text. Discrepancies between the studies selected by the two reviewers were resolved by consensus. When necessary, a third reviewer was consulted.

### Data extraction and quality assessment

Two reviewers working independently extracted data on study characteristics, interventions, patient characteristics at baseline, and outcomes for the study populations of interest for the final list of selected eligible studies. Study characteristics extracted included study design, inclusion and exclusion criteria, active treatment duration, and study quality items. Intervention characteristics extracted included dose, frequency of administration, duration, and concomitant/background therapies. Patient baseline characteristics extracted included age, sex, ethnicity, baseline prolactin levels (for males, females, and both sexes combined), previous antipsychotic use, age of schizophrenia onset, years since diagnosis, age at diagnosis, and schizophrenia subtype. Baseline disease severity scores from the Positive and Negative Syndrome Scale (PANSS) and the Brief Psychiatric Rating Scale (BPRS) were also extracted. Discrepancies between reviewers were resolved by involving a third reviewer and coming to a consensus.

For included RCTs, the quality of individual trials was assessed using the Risk of Bias instrument endorsed by the Cochrane Collaboration [18]. This instrument is used to evaluate 7 key domains: sequence generation, allocation concealment, blinding of participants and personnel, blinding of outcome assessors, incomplete outcome data, selective outcome reporting, and other sources of bias. For included observational studies, the quality of individual studies was assessed using the Newcastle-Ottawa Scale (NOS) [19]. This instrument is used to assess selection of patients, comparability of cohorts, and adequacy of outcomes and exposures.

### Data synthesis and analysis

Data across studies were compared to identify possible trends between treatments with regards to incidence of adverse events and changes in prolactin levels from baseline in a narrative review. RCT and observational evidence were examined separately. Data for both study designs were extracted as either binary (prolactin-related adverse events) or continuous (change in prolactin levels). Data on change in prolactin levels were extracted for both males and females separately as well as one aggregate group (males and females together). In the absence of estimates for mean change form baseline in prolactin levels, this was calculated where baseline prolactin levels and endpoint prolactin levels were presented by subtraction of the means (calculation of the confidence interval by pooling of the standard errors of the two estimates). Where mean and confidence intervals of change in prolactin levels were provided for two of the three groups, both mean and standard error (which was used to calculate the confidence interval) were back-calculated to obtain estimates for the third group (for instance, if mean change in prolactin levels for both males and females aggregated was presented as well as data on males, this could be used to back-calculate estimates for females). Pooled proportions were calculated where multiple studies assessed the same treatment regimen and were calculated as the back-transformation of the weighted mean of the transformed proportions, as outlined by Miller 1978 [20]. Finally, forest plots were created depicting extracted data from RCTs (not created for data from observational studies due to expected heterogeneity in treatment duration between studies). These depict differences in mean change from baseline in prolactin levels between treatments. Forest plots and pooled proportions summarizing relevant outcome data were created in StatsDirect (Version 2.8.0).

## Results

**Fig. 1:**
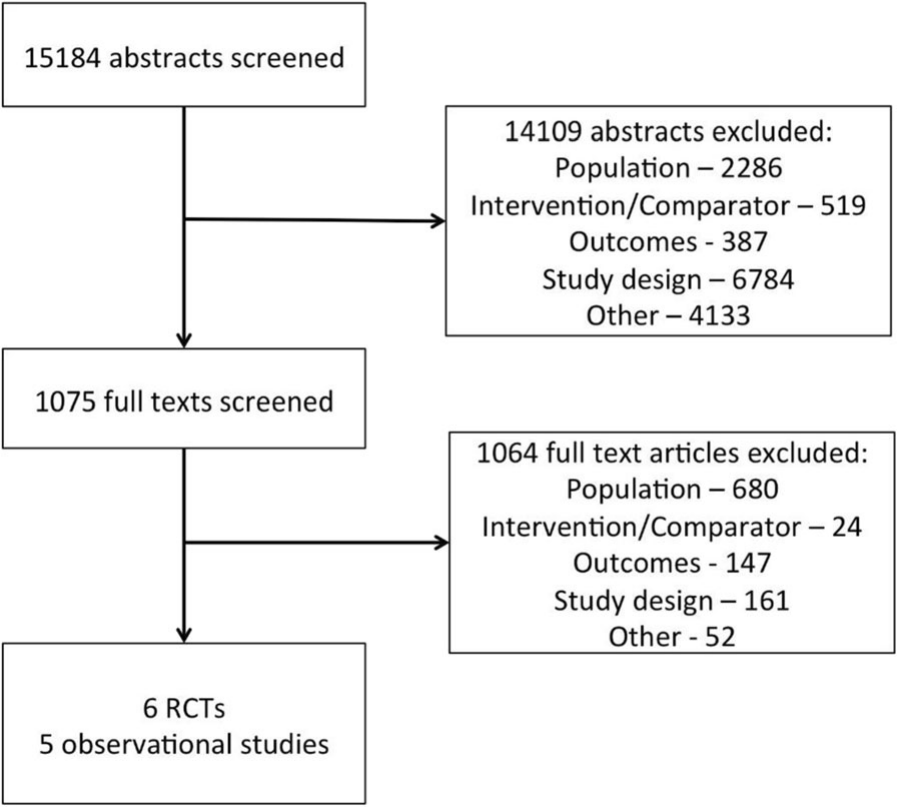
PRISMA study flow diagram

Of the 15,184 abstracts identified, 1,075 were evaluated at the full-text level. During the abstract screening phase, the most common reasons for exclusion were: studies were systematic reviews (classified as excluded for study design); studies were duplicates (classified as excluded for other); and studies assessed a population over 18 years of age (classified as excluded for population). The most common reason for exclusion in the full-text screening phase was a population over 18 years of age (classified as excluded for population). Eleven eligible studies were included, consisting of six RCTs and five non-randomized observational studies. Study flow is presented in Fig. 1.

### Evidence from RCTs

All six included RCTs evaluated the use of SGAs [21–26]. The interventions assessed by these studies include four dose ranges of risperidone (1–3 mg/day, 4–6 mg/day, 1.5-6 mg/day, and 0.15–0.6 mg/day) [23, 26], two doses of quetiapine (400 mg/day and 800 mg/day) [21], two doses of aripiprazole (10 mg/day and 30 mg/day) [22], olanzapine 2.5–20 mg/day [24], and three doses of paliperidone (1.5 mg/day, 3–6 mg/day, and 6–12 mg/day) [25]. All RCTs, with the exception of Haas et al. 2009b, included a placebo control arm [26]. Characteristics of included RCTs are presented in Table 1.

**Table 1:** Characteristics of included studies

Study	Diagnosis (criteria for diagnosis)	Treatment(s)	Treatment duration, weeks	Inclusion criteria
Randomized controlled trials				
Findling et al. 2012 [21]	Schizophrenia (DSM-IV-TR + K-SADS-PL)	Quetiapine 400 mg/day Quetiapine 800 mg/day Placebo	6	Inpatients or outpatients aged 13–17 years; PANSS ≥ 60
Findling et al. 2008 [22]	Schizophrenia (DSM-IV)	Aripiprazole 10 mg/day Aripiprazole 30 mg/day Placebo	6	Aged 13–17 years; PANSS ≥ 70
Haas et al. 2009a [23]	Schizophrenia (DSM-IV + K-SADS-PL)	Risperidone 1–3 mg/day Risperidone 4–6 mg/day Placebo	6	Aged 13–17 years; PANSS 60–120
Haas et al. 2009b [26]	Schizophrenia (DSM-IV + K-SADS-PL)	Risperidone 1.5–6 mg/day Risperidone 0.15–0.6 mg/day	8	Aged 13–17 years; PANSS 60–120
Kryzhanovskaya et al. 2009 [24]	Schizophrenia (DSM-IV-TR)	Olanzapine 2.5–20 mg/day Placebo	6	Aged 13–17 years; BPRS ≥ 35
Singh et al. 2011 [25]	Schizophrenia (DSM-IV + K-SADS-PL)	Paliperidone 1.5 mg/day Paliperidone 3–6 mg/day Paliperidone 6–12 mg/day Placebo	6	Aged 12–17 years; Weight ≥ 29 kg; PANSS 60–120
Observational studies				
Duval et al. 2008 [27]	Schizophreniform disorder (DSM-IV)	Risperidone 1–6 mg/day	3	Aged 14–18 years
Kumra et al. 2008 [28]	Schizophrenia, schizoaffective disorder (K-SADS-PL)	Clozapine 25–900 mg/day Olanzapine 30 mg/day	12	Aged 10–18 years
Pandina et al. 2012 [29]	Schizophrenia (DSM-IV + K-SADS-PL)	Risperidone 2–6 mg/day^a^	24 or 48^a^	Aged 13–17 years; PANSS 40–120
Ruan et al. 2010 [30]	Schizophrenia (DSM-IV-TR)	Risperidone 25–50 mg biweekly	24	Aged 13–18 years; PANSS ≤ 80
Schimmelmann et al. 2007 [31]	Schizophrenia, schizoaffective and schizophreniform disorder (DSM-IV)	Quetiapine 200–800 mg/day	12	Aged 12–17 years; PANSS ≥ 60

*BPRS* Brief psychiatric rating scale, *DSM* Diagnostic and Statistical Manual of Mental Disorders, *K-SADS-PL* Kiddie Schedule for Affective Disorders and Schizophrenia for School-Age Children – Present and Lifetime Version, *PANSS* Positive and negative syndrome scale, *TR* text revision

^a^Patients were allocated to three treatment arms as treatment-naïve, treatment-experienced, and open-label treatment. Patients in the treatment-naïve and treatment-experienced arms were evaluated at 24 weeks and patients in the open-label arm were evaluated at 48 weeks

Treatment duration for most studies was 6 weeks, with the exception of Haas et al. 2009b which reported outcomes after an 8 week treatment duration [26]. All RCTs included adolescent patients, with age at enrollment restrictions varying between 12 and 18 years. Three RCTs only included patients with a PANSS score between 60 and 120 [23, 25, 26] while one study included any patients with a PANSS score of 60 or above [21] and one study included patients with a PANSS score of 70 or above [22].

#### Baseline patient characteristics

Patient baseline characteristics are presented in Table 2. Baseline characteristics were generally well balanced, both between treatment arms and trials. Mean age varied between 15.1 and 16.3 years for all included RCTs and the percentage of males varied between 45 % and 73 %. The distribution of males between study arms differed significantly for two trials, one reporting on the effects of aripiprazole, with percentages between 45 % and 64 % for the three arms [22], and the other reporting on paliperidone, with percentages between 45 % and 70 % for the four trial arms [25]. The majority of the study populations consisted of Caucasians, with all other races and ethnicities making up less than one quarter of each study arm in most studies. In the study by Haas et al. 2009a, however, Asian patients comprised over a third of the study population.

**Table 2:** Summary of baseline characteristics

Study	N (ITT)	Treatment	Age, mean (SD)	Male, n (%)	Caucasian, n (%)	Baseline prolactin, both sexes, (ng/mL), mean (SD)	Baseline prolactin, males, (ng/mL), mean (SD)	Baseline prolactin, females, (ng/mL), mean (SD)	Previous antipsychotic use, n (%)	Proportion diagnosed as paranoid schizophrenia, n (%)	Baseline PANSS score, mean (SD)
Randomized controlled trials											
Findling et al. 2012 [21]	73	Quetiapine 400 mg/day	15.5 (1.3)	43 (58.9)	45 (61.6)	20.8 (17.0)	NR	NR	NR	NR	96.2 (17.7)
	74	Quetiapine 800 mg/day	15.5 (1.3)	44 (59.5)	44 (59.5)	18.1 (20.1)	NR	NR	NR	NR	97.0 (15.3)
	73	Placebo	15.3 (1.4)	42 (57.5)	46 (63.0)	28.7 (29.1)	NR	NR	NR	NR	96.7 (18.0)
Findling et al. 2008 [22]	100	Aripiprazole 10 mg/day	15.6 (1.3)	45 (45.0)	5]4 (54.0)	NR	NR	NR	53 (53.0)	NR	93.7 (15.7)
	102	Aripiprazole 30 mg/day	15.4 (1.4)	65 (63.7)	62 (60.8)	NR	NR	NR	47 (46.1)	NR	94.9(15.5)
	100	Placebo	15.4 (1.4)	61 (61.0)	64 (64.0)	NR	NR	NR	46 (46.0)	NR	95.0(15.5)
Haas et al. 2009a [23]	55	Risperidone 1–3 mg/day	15.7 (1.3)	30 (54.5)	33 (60.0)	29.6 (38.2)	21.6 (15.2)	39.2 (53.0)	NR	38 (69.1)	NR
	51	Risperidone 4–6 mg/day	15.7 (1.3)	37 (72.5)	24 (47.1)	24.1 (23.0)	22.7 (19.9)	27.9 (28.3)	NR	34 (66.7)	NR
	54	Placebo	15.5 (1.4)	35 (64.8)	27 (50.0)	22.6 (23.3)	21.5 (21.1)	24.6 (25.9)	NR	38 (70.4)	NR
Haas et al. 2009b [26]	125	Risperidone 1.5–6 mg/day	15.6 (1.3)	65 (52.0)	104 (83.2)	NR	NR	NR	NR	83 (66.4)	96.4 (15.4)
	132	Risperidone 0.15–0.6 mg/day	15.6 (1.3)	80 (60.6)	111 (84.9)	NR	NR	NR	NR	92 (69.7)	93.3 (14.1)
Kryzhanovskaya et al. 2009 [24]	72	Olanzapine 2.5–20 mg/day	16.1 (1.3)	51 (70.8)	52 (72.2)	15.6 (12.3)	NR	NR	51 (70.8)	NR	95.3 (14.1)
	35	Placebo	16.3 (1.6)	24 (68.6)	25 (71.4)	18.7 (16.2)	NR	NR	30 (85.7)	NR	95.5 (14.1)
Singh et al. 2011 [25]	54	Paliperidone 1.5 mg/day	15.1 (1.5)	30 (55.6)	35 (64.8)	25.1 (32.8)	14.7 (14.5)	38.0 (45.5)	47 (87.0)	NR	91.6 (12.5)
	48	Paliperidone 3–6 mg/day	15.3 (1.6)	31 (64.6)	34 (70.8)	26.6 (43.2)	16.1 (1.8)	45.67 (71.0)	44 (91.7)	NR	90.6 (14.0)
	47	Paliperidone 6–12 mg/day	15.5 (1.6)	33 (70.2)	32 (68.1)	20.8 (22.3)	18.2 (13.7)	26.88 (33.9)	40 (85.1)	NR	91.5 (13.9)
	51	Placebo	15.7 (1.4)	23 (45.1)	35 (68.6)	24.5 (30.5)	13.6 (13.6)	33.43 (38.5)	48 (94.1)	NR	90.6 (12.1)
Observational studies											
Duval et al. 2008 [27]	16	Risperidone 1–6 mg/day	15.7 (1.3)	10 (62.5)	16 (100.0)	16 (9)	NR	NR	NR	NR	NR
Kumra et al. 2008 [28]	14	Clozapine 25–900 mg/day	15.3 (2.3)	5 (35.7)	1 (7.1)	NR	NR	NR	14 (100.0)	NR	NR
	19	Olanzapine 30 mg/day	15.6 (1.7)	11 (57.9)	5 (26.3)	NR	NR	NR	19 (100.0)	NR	NR
Pandina et al. 2012 [29]	48	Risperidone 2–6 mg/day (treatment-naïve)	15.4 (1.4)	30 (62.5)	25 (52.1)	17.0 (20.4)	18.6 (24.7)	14.3 (6.7)	NR	33 (68.8)	84.7 (16.8)
	292	Risperidone 2–6 mg/day (treatment-experienced)	15.5 (1.7)	178 (61.0)	218 (74.7)	53.4 (33.9)	40.4 (26.3)	73.6 (43.0)	292 (100.0)	195 (66.8)	72.1 (19.4)
	50	Risperidone 2–6 mg/day (open-label)	15.5 (1.4)	30 (60.0)	42 (84.0)	73.6 (44.9)	55.8 (25.3)	100.3 (62.3)	NR	35 (70.0)	83.9 (13.5)
Ruan et al. 2010 [30]	31	Risperidone 25–50 mg biweekly	15.9 (3.3)	13 (41.9)	NR	54.8 (17.7)	NR	NR	NR	24 (77.4)	57.8 (1.8)
Schimmelmann et al. 2007 [31]	56	Quetiapine 200–800 mg/day	15.9 (1.3)	38 (67.9)	47 (83.9)	15.9 (23.3)	12.6 (6.3)	22.9 (41.4)	13 (23.2)	NR	91.5 (17.2)

*ITT* Intention to treat, *NR* Not reported, *SD* Standard deviation

Of the three RCTs reporting on previous antipsychotic use, two reported percentages of patients with previous antipsychotic use above 70 % in all arms [24, 25] and one reported previous antipsychotic use of 46 %, 53 %, and 46 % in the placebo arm, aripiprazole 10 mg/day arm, and aripiprazole 30 mg/day arm, respectively [22].

Three RCTs reported mean age of onset of symptoms related to schizophrenia, which varied between 12.5 and 14.2 years of age [22, 24, 26]. Three RCTS reported mean age at diagnosis of schizophrenia, which was also similar across trials, varying from 12.5 years of age to 15.3 years of age [23, 25, 26]. In addition, two three-arm studies reported mean years since schizophrenia diagnosis, which was similar in all three arms for both studies [21, 22]. Three RCTs reported the proportion of patients diagnosed under each schizophrenia subtype [21, 23, 26]. In all of these studies, the majority of patients were diagnosed with paranoid schizophrenia, followed by undifferentiated schizophrenia and disorganized schizophrenia [23, 26]. No study reported age at first psychosis.

Baseline disease severity assessments were consistent across studies. Most RCTs reported baseline disease severity using the PANSS, with mean values varying from 90.6 to 97.0 [21, 22, 24–26]. One RCT reported mean baseline BPRS scores of 50.3 and 50.1 for the olanzapine 2.5–20 mg/day and placebo arms, respectively [24].

Two studies reported overall population baseline prolactin levels, with Findling et al. (2012) reporting mean baseline values between 18.1 ng/mL and 28.7 ng/mL and Kryzhanovskaya et al. (2009) reporting mean baseline values between 15.6 ng/mL and 18.7 ng/mL [21, 24]. Additionally, two trials reported baseline prolactin levels for both males and females separately. For males, Haas et al. (2009a) reported mean baseline values from 21.5 ng/mL and 22.7 ng/mL, while Singh et al. (2011) reported values between 13.6 ng/mL and 18.2 ng/mL [23, 25]. Baseline prolactin levels were higher in females, but similar across studies, as Haas et al. (2009a) reported values between 24.6 ng/mL and 39.2 ng/mL, while Singh et al. (2011) reported values between 26.9 ng/mL and 45.7 ng/mL [23, 25]. Overall population baseline prolactin levels were calculated for these two studies.

#### Outcomes

##### Prolactin-related adverse events

Prolactin-related adverse events were sparsely reported (see Table 3). Three RCTs reported overall prolactin-related adverse events, gynecomastia/galactorrhea, and amenorrhea/dysmenorrhea in patients treated with varying doses of aripiprazole and risperidone [22, 23, 26]. In two studies, which assessed aripiprazole and risperidone, no cases of prolactin-related adverse events were reported [22, 23]. A study comparing doses of risperidone of 1.5–6 mg/day and 0.15–0.6 mg/day reported seven events (5.6 % of study patients) and two events (1.5 %), respectively [26]. Specifically, investigators reported three (2.4 %) and two (1.5 %) cases of gynecomastia/galactorrhea in the risperidone 1.5–6 mg/day and risperidone 0.15–0.6 mg/day arms, respectively, as well as a single case (1.7 % of female patients) of amenorrhea/dysmenorrhea in the risperidone 1.5–6 mg/day arm.

**Table 3:** Prolactin-related adverse events

Study	N (ITT)	Treatment	Prolactin-related adverse events, n (%)	Gynecomastia/ galactorrhea, n (%)	Amenorrhea/ dysmenorrhea, n (%)	Impotence/decreased libido, n (%)
Randomized controlled trials						
Findling et al. 2012 [21]	73	Quetiapine 400 mg/day	NR	NR	NR	NR
	74	Quetiapine 800 mg/day	NR	NR	NR	NR
	73	Placebo	NR	NR	NR	NR
Findling et al. 2008 [22]	100	Aripiprazole 10 mg/day	0 (0.0)	NR	NR	NR
	102	Aripiprazole 30 mg/day	0 (0.0)	NR	NR	NR
	100	Placebo	0 (0.0)	NR	NR	NR
Haas et al. 2009a [23]	55	Risperidone 1–3 mg/day	0 (0.0)	NR	NR	NR
	51	Risperidone 4–6 mg/day	0 (0.0)	NR	NR	NR
	54	Placebo	0 (0.0)	NR	NR	NR
Haas et al. 2009b [26]	125	Risperidone 1.5–6 mg/day	7 (5.6)	3 (2.4)	1 (1.0)	NR
	132	Risperidone 0.15–0.6 mg/day	2 (1.5)	2 (1.5)	0 (0.0)	NR
Kryzhanovskaya et al. 2009 [24]	72	Olanzapine 2.5–20 mg/day	NR	NR	NR	NR
	35	Placebo	NR	NR	NR	NR
Singh et al. 2011 [25]	54	Paliperidone 1.5 mg/day	NR	NR	NR	NR
	48	Paliperidone 3–6 mg/day	NR	NR	NR	NR
	47	Paliperidone 6–12 mg/day	NR	NR	NR	NR
	51	Placebo	NR	NR	NR	NR
Observational studies						
Duval et al. 2008 [27]	16	Risperidone 1–6 mg/day	NR	NR	NR	NR
Kumra et al. 2008 [28]	14	Clozapine 25–900 mg/day	NR	NR	NR	NR
	19	Olanzapine 30 mg/day	NR	NR	NR	NR
Pandina et al. 2012^a^ [29]	48	Risperidone 2–6 mg/day (treatment-naïve)	36 (9.3)	14 (3.6)	3 (1.0)	1 (<0.5)
	292	Risperidone 2–6 mg/day (treatment-experienced)				
	50	Risperidone 2–6 mg/day (open-label)				
Ruan et al. 2010 [30]	31	Risperidone 25–50 mg biweekly	5 (16.1)	2 (6.5)	3 (9.7)	NR
Schimmelmann et al. 2007 [31]	56	Quetiapine 200–800 mg/day	NR	3 (5.4)	4 (7.1)	9 (16.1)

*ITT* Intent to treat, *NR* Not reported

^a^Incidence of adverse events reported across all treatment arms

##### Change in prolactin levels: Change from baseline, treatment-placebo comparison

**Fig. 2:**
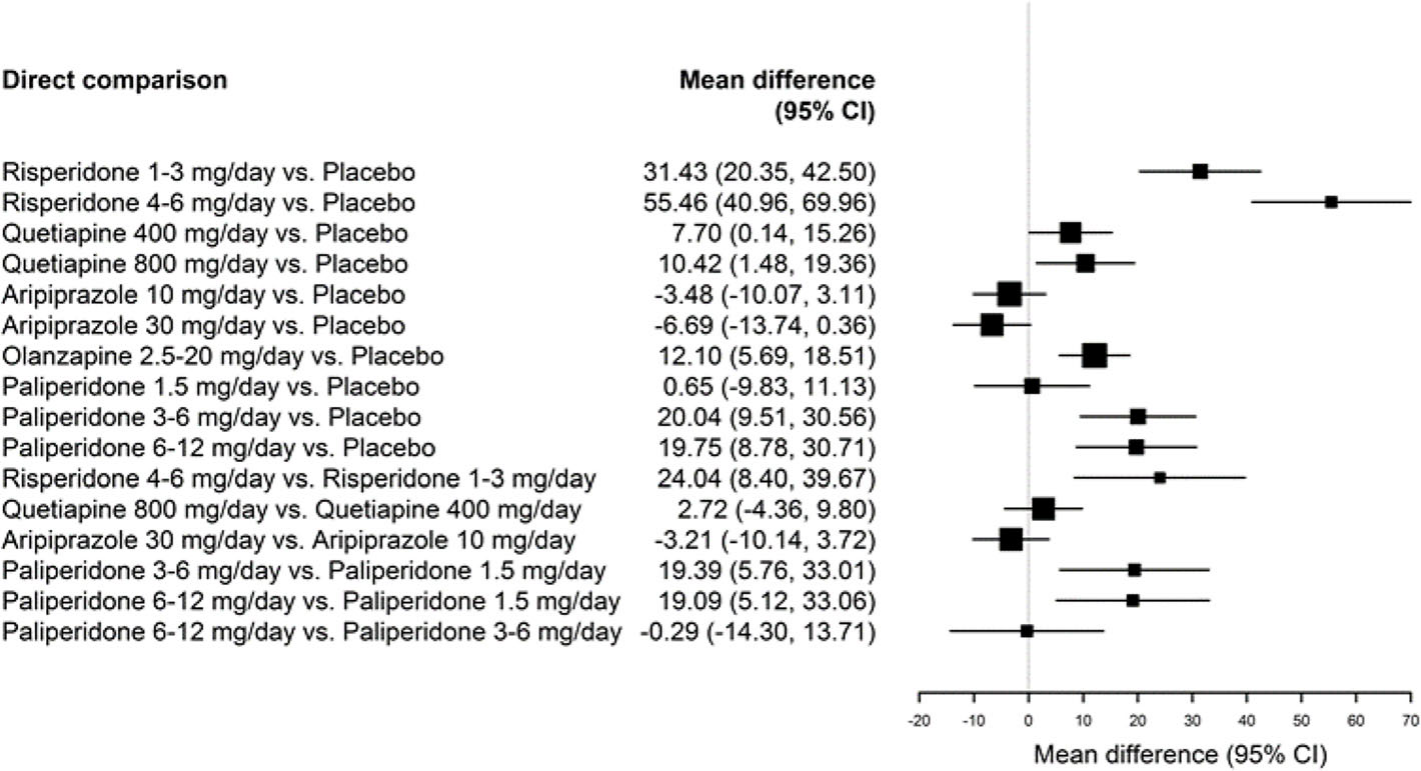
Forest plot of mean change from baseline for SGAs presented in RCTs, overall population

Five RCTs provided data for mean change in prolactin levels for the overall population (see Table 4) [21–25]. Mean changes from baseline compared to placebo for each intervention in the overall population are presented in Fig. 2.

**Table 4:** Changes from baseline in prolactin

Study	Treatment	Time point, weeks	Prolactin change, both sexes (ng/mL), mean (SD)	Prolactin change, males (ng/mL), mean (SD)	Prolactin change, females (ng/mL), mean (SD)
Randomized controlled trials					
Findling et al. 2012 [21]	Quetiapine 400 mg/day	6	−10.6 (16.1)	−9.22 (14.4)	−12.4 (18.5)
	Quetiapine 800 mg/day	6	−7.8 (26.5)	−3.7 (11.6)	−14.0 (39.1)
	Placebo	6	−18.3 (28.8)	−6.53 (15.1)	−33.9 (34.9)
Findling et al. 2008 [22]	Aripiprazole 10 mg/day	6	−11.9 (23.3)	NR	NR
	Aripiprazole 30 mg/day	6	−15.1 (26.9)	NR	NR
	Placebo	6	−8.5 (24.2)	NR	NR
Haas et al. 2009a [23]	Risperidone 1–3 mg/day	6	25.5 (33.5)	16 (23.7)	36.9 (41.3)
	Risperidone 4–6 mg/day	6	49.5 (46.9)	26.4 (28.5)	77.3 (60.8)
	Placebo	6	−5.9 (24.9)	−3.2 (24.8)	−9.2 (24.1)
Haas et al. 2009b [26]	Risperidone 1.5–6 mg/day	8	NR	NR	NR
	Risperidone 0.15–0.6 mg/day	8	NR	NR	NR
Kryzhanovskaya et al. 2009 [24]	Olanzapine 2.5–20 mg/day	6	8.8 (17.9)	NR	NR
	Placebo	6	−3.3 (14.8)	NR	NR
Singh et al. 2011 [25]	Paliperidone 1.5 mg/day	6	3.3 (36.0)	3.6 (19.1)	2.9 (48.6)
	Paliperidone 3–6 mg/day	6	22.7 (34.1)	22.8 (30.1)	22.4 (38.7)
	Paliperidone 6–12 mg/day	6	22.4 (35.5)	21.3 (31.1)	24.9 (42.0)
	Placebo	6	2.7 (15.2)	0.6 (9.4)	4.4 (18.3)
Observational studies					
Duval et al. 2008 [27]	Risperidone 1–6 mg/day	3	47.9 (23.6)	NR	NR
Kumra et al. 2008 [28]	Clozapine 25–900 mg/day	12	NR	NR	NR
	Olanzapine 30 mg/day	12	NR	NR	NR
	Clozapine 25–900 mg/day	24	NR	NR	NR
	Olanzapine 30 mg/day	24	NR	NR	NR
Pandina et al. 2012 [29]	Risperidone 2–6 mg/day (treatment-naïve)	24	66.8 (41.8)	29.1 (32.6)	83.4 (44.7)
	Risperidone 2–6 mg/day (treatment-experienced)	24	−11.6 (43.3)	−6.2 (22.2)	−14.0 (49.3)
	Risperidone 2–6 mg/day (open-label)	48	6.8 (35.2)	3.7 (28.5)	11.5 (43.3)
Ruan et al. 2010 [30]	Risperidone 25–50 mg biweekly	24	−13.1 (17.1)	NR	NR
Schimmelmann et al. 2007 [31]	Quetiapine 200–800 mg/day	12	−1.4 (21.1)	−4.5 (5.7)	3.2 (21.7)

*NR* Not reported, *SD* Standard deviation

Compared to baseline, both doses of quetiapine and aripiprazole resulted in decreased prolactin levels. When compared to placebo, these changes were significantly greater compared to baseline for quetiapine but not for aripiprazole.

Both doses of risperidone, olanzapine, and paliperidone 3–5 mg/day and 6–12 mg/day were found to increase prolactin levels from baseline. These changes also reflected statistically significant increases when compared to placebo. Paliperidone 1.5 mg/day was not associated with a statistically significant change.

**Fig. 3:**
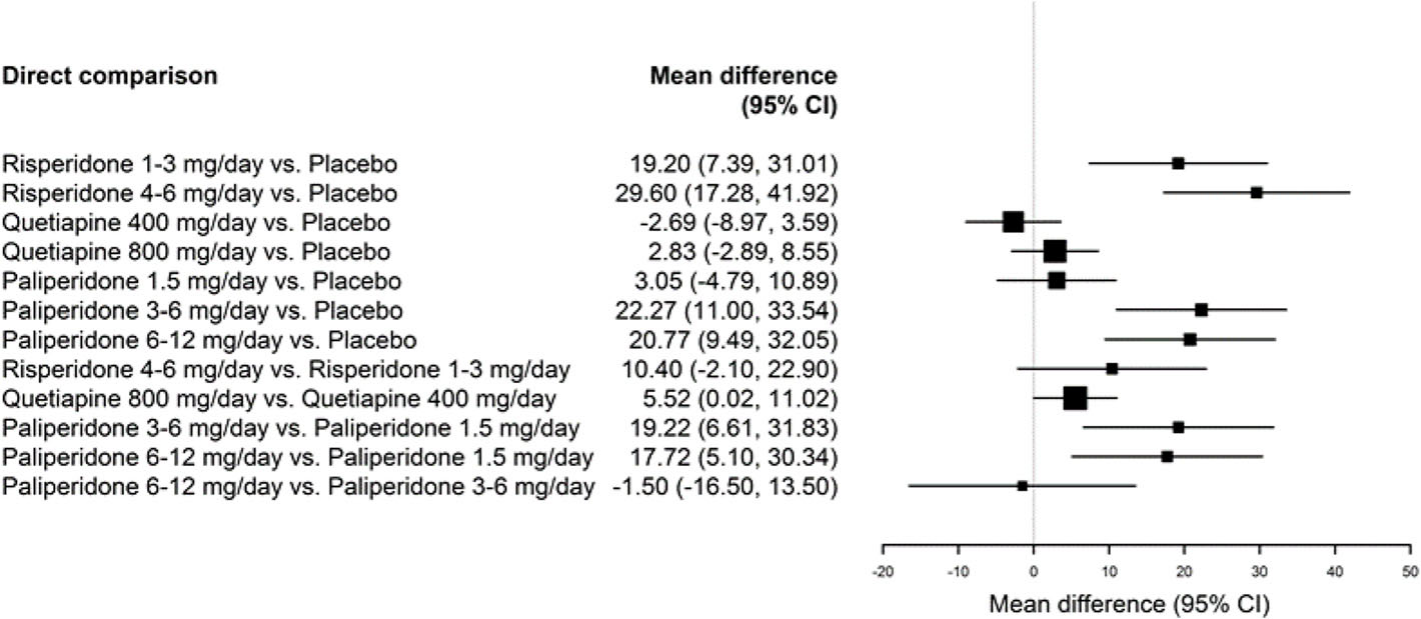
Forest plot of mean change from baseline for SGAs presented in RCTs, male population only

**Fig. 4:**
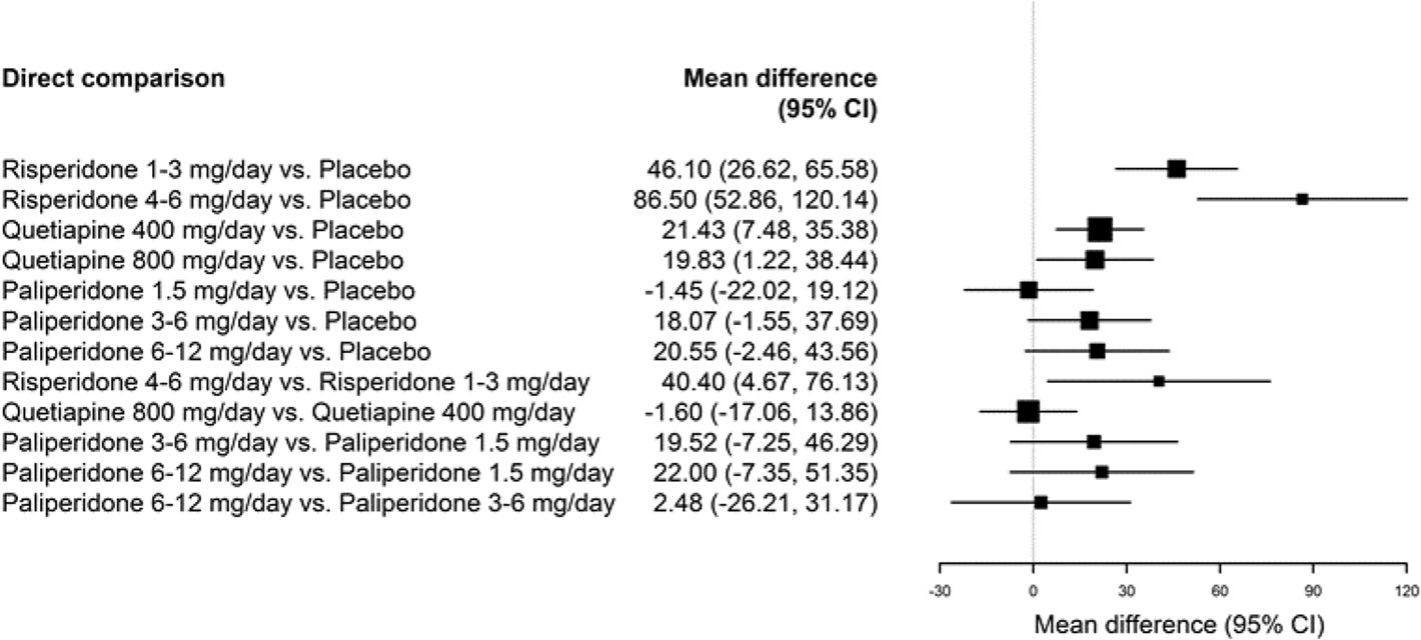
Forest plot of mean change from baseline for SGAs presented in RCTs, female population only

Three RCTs reported changes in serum prolactin levels stratified by sex [21, 23, 25]. When evaluating the change from baseline for each of the included SGAs, trends reflected those observed in the overall populations for both males and females. However, the change from baseline for females treated with any dose range of risperidone was higher than males treated with the same dose. Figures 3 and 4 present the mean changes from baseline compared to placebo for males and females, respectively. Compared to placebo, the change from baseline for females treated with either dose of quetiapine resulted in a statistically significant difference. This change was not found to be significant in males. Compared to placebo, both males and females were found to experience significant increases when treated with risperidone, though this increase was more marked in female patients treated with risperidone 4–6 mg/day. Conversely, the change from baseline compared to placebo was significant for males, but not females, treated with paliperidone 3–6 mg/day and 6–12 mg/day.

##### Change in prolactin levels: Change from baseline, treatment-treatment comparison

Figure 2 presents comparisons for change from baseline in prolactin levels between treatments for the overall population. Risperidone 4–6 mg/day reported a greater increase in prolactin levels when compared with the lower dose of 1–3 mg/day. Similarly, both paliperidone 3–6 mg/day and 6–12 mg/day reported a greater increase in prolactin levels when compared with paliperidone 1.5 mg/day. However, the difference between paliperidone 6–12 mg/day and 3–6 mg/day was not statistically significant. Further, no statistically significant differences were reported for the comparisons between quetiapine 400 mg/day and 800 mg/day or between aripiprazole 30 mg/day and 10 mg/day.

Figures 3 and 4 present comparisons of change in prolactin levels from baseline between treatments for the male and female populations, respectively. The comparison between risperidone at 4–6 mg/day and 1–3 mg/day was significant in the female population, but not in the male population, with the higher dose producing a greater change from baseline for both populations. Similarly, comparisons between quetiapine 800 mg/day and 400 mg/day, paliperidone 6–12 mg/day and 1.5 mg/day, and paliperidone 6–12 mg/day and 1.5 mg/day were statistically significant in the male population, but not in the female population, with greater increases observed in the treatments with the higher dose. The comparison between paliperidone 6–12 mg/day and paliperidone 3–6 mg/day was not significant for either patient population.

### Evidence from non-randomized, observational studies

Five observational studies were identified which assessed patient experiences with SGAs [27–31]. The interventions represented in this set of literature included three doses of risperidone (1–6 mg/day, 2–6 mg/day, 25–50 mg biweekly) [27, 29, 30], quetiapine 200–800 mg/day [31], clozapine 25–900 mg/day [28], and olanzapine 30 mg/day [28]. Characteristics of included observational studies are presented in Table 1.

Two of the observational studies restricted enrollment to patients with a diagnosis of only schizophrenia [29, 30]. The remaining studies represented patients with schizophrenia, schizoaffective disorder, and schizophreniform disorder [27, 28, 31]. Three of the observational studies also specified disease severity in the inclusion criteria, with PANSS scores restricted to between 40 and 120 [29], 80 or below [30], and 60 or above [31].

Treatment durations varied widely, with one observational study reporting on patient outcomes after 3 weeks of treatment [27], two studies after 12 weeks [28, 31], one study after 24 weeks [30], and one three-arm study after 24 weeks for two arms and 48 weeks for the third arm [29]. All observational studies included adolescent patients with ages varying between 10 and 18 years.

One study, published by Pandina and colleagues (2012), adopted a non-conventional comparative observational study methodology [29]. This three-arm study was designed to include one arm with 24 weeks prior treatment experience with risperidone 2–6 mg/day (treatment-experienced arm), one arm with no risperidone experience (treatment-naïve arm), and one arm with varying levels of treatment experience prior to study treatment (open-label arm). At enrollment, patients received 24 weeks open-label treatment with risperidone 2–6 mg/day, thus resulting in one arm with 48 weeks of treatment experience and two arms with 24 weeks of treatment experience.

#### Baseline patient characteristics

Patient baseline characteristics are presented in Table 2. Mean ages were homogenous across studies, varying between 15.3 and 15.9 years. However, the distribution of males and females varied between trials, with percentages of males in each study arm ranging from 36 % to 68 %. Race was reported in four observational studies [27–29, 31]. In two cases, patient populations were comprised either primarily or entirely of Caucasians [27, 31]. Imbalances were reported in the three-arm study, with the percentage of Caucasian patients ranging from 52 % to 84 % across arms [29]. The study by Kumra et al. (2008) comprised a mixed race population with African-Americans representing the largest percentage of patients at 36 % and 47 % across treatment arms [28].

Where reported, previous antipsychotic treatment experience varied from 100 % of patients [28, 29] to less than 25 % [31]. Similarly, disease severity was not consistent across the included studies. As assessed by the PANSS, mean reported scores varied from 57.8 to 91.5. A single study reported mean age of schizophrenia symptoms of 12.7 and 11.7 for the two treatment arms [32]. Mean age of diagnosis of schizophrenia or schizophrenia spectrum disorder was reported in a single study, with ages varying between 14.8 and 15.1 across study arms [29]. Two observational studies reported schizophrenia subtype and in both cases the majority of patients were diagnosed with paranoid schizophrenia [29, 30]. Overall mean baseline prolactin levels varied between studies from a minimum of 15.9 ng/mL to a maximum of 73.6 ng/mL. In two observational studies, baseline prolactin levels for males and females were available separately and differed between sexes [29, 31]. Pandina et al. (2012) reported baseline prolactin levels for males of 18.6 ng/mL, 40.4 ng/mL, and 55.8 ng/mL and baseline prolactin levels for females of 14.3 ng/mL, 73.6 ng/mL, and 100.3 ng/mL for the treatment-naïve, treatment-experienced, and open-label arms, respectively [29]. This difference was not as marked, but still different, in the paper by Schimmelmann and colleagues (2007), which reported levels of 12.6 ng/mL and 22.9 ng/mL for males and females, respectively [31].

#### Outcomes

Outcomes were typically reported after 12 or 24 weeks of treatment. Prolactin-related adverse events were reported in three studies; two reporting on patient experience with varying levels of risperidone [29, 30] and one with quetiapine 200–800 mg daily [31]. Pandina et al. (2012) reported 36 (9 %) prolactin-related adverse events, 14 cases (3.6 %) of gynecomastia/galactorrhea, 3 cases (1.0 %) of amenorrhea/dysmenorrhea, and a single case (<0.5 %) of impotence or decreased libido across study arms. These adverse events were not reported by treatment arm, which differed with regards to prior treatment experience and treatment duration. In a study of patients treated biweekly with 25–50 mg of risperidone for 24 weeks, 5 cases (16 %) of prolactin-related adverse events, comprising 2 cases (6.5 %) of gynecomastia/galactorrhea and 3 cases (9.7 %) of amenorrhea/dysmenorrhea, were reported [30]. A single study assessing quetiapine reported 3 cases (5.4 %) of gynecomastia/galactorrhea, 4 cases (7.1 %) of amenorrhea/dysmenorrhea, and 9 cases (16.1 %) of impotence or decreased libido [31].

Mean change from baseline in prolactin levels is presented in Table 4. Mean changes were available in four studies; one reporting on quetiapine [31] and three on risperidone [27, 29, 30]. Patients treated with quetiapine 200–800 mg/day experienced a mean decrease of 1.4 ng/mL (standard deviation [SD] 21.1) [31]. Changes in patients treated with risperidone varied considerably, from a mean increase of 66.8 ng/mL (SD 41.8) to a mean decrease of 13.1 ng/mL (SD 17.1).

Change from baseline stratified by sex was available in two studies [29, 31]. In the first study, male patients treated with quetiapine 200–800 mg/day were found to have a mean decrease of 4.5 ng/mL (SD 5.7), while females experienced an increase of 3.2 ng/mL (SD 21.7) [31]. In the second study, male patients treated with risperidone 2–6 mg/day who were previously treatment-naïve were found to have a mean increase of 29.1 ng/mL (SD 32.6), whereas treatment-experienced patients were found to decrease levels by 6.2 ng/mL (SD 22.2). Female patients experienced larger changes from baseline, with treatment-naïve patients reporting mean increases of 83.4 ng/mL (SD 44.7) and treatment-experienced patients reporting a mean decrease of 14.0 ng/mL (49.3). In the open-label arm of this risperidone study, which consisted of patients with varying levels of treatment experience, males were reported to have a mean increase of 3.7 ng/mL (SD 28.5) while females reported a mean increase of 11.5 ng/mL (SD 43.3) [29].

### Risk of bias assessment

The Cochrane risk of bias tool was used to assess the quality of included RCTs [18]. A lack of reporting on sequence generation [21–24] and allocation concealment [22–24, 26], as well as a lack of completeness for primary outcome data [22] were raised as causes for concern in a number of trials. Other sources of bias, which included lack of reporting on specific baseline characteristics (prolactin levels) and prior treatment experience, were deemed high in three studies [22–24]. Overall, included RCTs were deemed to have a minimal risk of bias. Results of this evaluation are available in the Additional file 1: Figure S1 and Additional file 2: Table S1.

The NOS was used to assess the quality of included observational studies [19]. All studies, with the exception of Ruan et al. 2010, studied populations of patients whose outcomes may be generalized to all pediatric patients with schizophrenia being treated with antipsychotics. Ruan et al. 2010 combined two study populations, one previously treated with olanzapine and the other previously treated with oral risperidone. However, baseline prolactin levels in these two groups were differed greatly (31.3 ng/mL [SD 11.3] and 87.4 ng/mL [SD 15.5] for patients with previous olanzapine and risperidone experience, respectively). As prolactin levels are measured biochemically, ascertainment of exposure presents minimal risk of bias. The included observational studies were considered comparable in that diagnoses and patient ages included were similar. However, differences in exclusion criteria for baseline disease severity and study durations did not allow for robust comparisons (one study had a follow-up time of less than four weeks, shorter than any of the included RCTs [27]). In all studies, loss-to-follow up was accounted for. Overall, this observational evidence presents a medium to high risk of bias.

## Discussion

This review was designed to evaluate the frequency of prolactin-related adverse events and to consolidate/quantify differences in prolactin level changes between antipsychotics in pediatric patients with schizophrenia and schizophrenia spectrum disorders. No definitive conclusions can be drawn regarding differences in prolactin-related adverse events between antipsychotics due to the paucity of data. However, analyses of mean change from baseline in prolactin levels demonstrated some differences between treatments.

A review of the evidence reported in RCTs found that, in analyses of both the overall population and the population stratified by patient sex, risperidone at 1–3 mg/day and 4–6 mg/day was associated with an increase in prolactin levels compared to baseline. This change was particularly marked in females. When compared to placebo, both doses of risperidone produced greater increases in prolactin levels. Risperidone 4–6 mg/day was found to produce greater increases than risperidone 1–3 mg/day in the overall population. However, this trend was not observed in males. A single-arm observational study by Duval et al. reported outcomes in patients treated with risperidone 1–6 mg/day. They found that prolactin levels increased, on average, in these patients after three weeks of treatment. This finding is in line with the findings in the RCT evidence. A three-arm study by Pandina et al. in patients treated with risperidone 2–6 mg/day reported outcomes at 24 weeks for two arms, one treatment-naïve and the other treatment-experienced, and 48 weeks for the remaining open-label arm. Patients in the treatment-experienced and open-label arms showed relatively little change in prolactin levels. However, this may be attributed to previous treatment experience. Patients in the treatment-naïve arm experienced increases in prolactin levels, with a more marked increase in females. However, these changes were accompanied by large dispersions. The findings in the treatment-naïve patients reflect those of the RCT evidence that risperidone is associated with an overall increase in prolactin levels. Differences in patient characteristics such as treatment-experience, which was not reported in the included RCTs, and time horizons may explain the contradictory nature between the observational and RCT evidence.

Paliperidone at doses of 3–6 mg/day and 6–12 mg/day, but not 1.5 mg/day, was associated with increases in prolactin levels between baseline and endpoint and when compared to placebo. When evaluating the evidence stratified by patient sex, the same trend was observed in males but not in females. Comparisons in the overall population between paliperidone doses suggest that paliperidone 3–6 mg/day and 6–12 mg/day both increase prolactin more than the 1.5 mg/day dose. However, this trend only held for males when results were stratified by patient sex. The difference in change in prolactin between paliperidone 6–12 mg/day and 3–6 mg/day was not statistically significant. No observational study was identified reporting on patients treated with paliperidone.

Changes in prolactin levels were significant for both doses of quetiapine and for aripiprazole in the RCT evidence, with mean decreases compared to baseline. When compared to placebo, quetiapine 800 mg/day reported an increase from baseline in prolactin levels over the study period. This change was statistically significant for females, but not for males. In the single observational study reporting on changes in patients treated with quetiapine, a decrease in prolactin levels in the overall and male only populations was reported, with a reported increase for the female population. It is of note that the dispersions reported with these values were wide. However, direct comparisons across these bodies of evidence must be made with caution, given that there a number of factors that may contribute in explaining the disparity of these results. For example, the doses included in the observational study varied considerably, from 200 mg/day to 800 mg/day, while patients in the RCT were treated with doses of 400 mg/day and 800 mg/day. Moreover, the observational study reported outcomes at 12 weeks, while the RCT reported outcomes at 6 weeks.

The findings of this review are generally in agreement with recent reviews on changes in prolactin levels relating to the use of antipsychotic medications [33, 34]. A 2012 review by Cohen et al. reported on short-term (3 to 12 week) outcomes from SGA use in child and adolescent patients for the management of a number of diagnoses, including schizophrenia, bipolar disorder, and conduct disorder. They identified four studies on the use of aripiprazole where patients were reported to have statistically significant decreases in prolactin levels compared to baseline. This is consistent with the findings of the current review. However, when changes from baseline were compared between aripiprazole and placebo in the current review, this decrease was not found to be statistically significant. Compared to placebo, Cohen reported that risperidone and olanzapine both produced increases in prolactin levels, which is consistent with the findings of the current review. Similarly, Leucht et al. reported increases in prolactin levels associated with the use of risperidone and paliperidone. However, the dose of paliperidone was not specified. In the current review, paliperidone was found to increase prolactin levels at the 3–6 mg/day and 6–12 mg/day dose ranges, but not in patients treated with the 1.5 mg/day regimen. Consistent with the current review, treatment with aripiprazole was not found to increase prolactin compared to placebo. Leucht et al. also found that quetiapine was not associated with increases when compared to placebo. In the current review, however, treatment with quetiapine at both the 400 mg/day and 800 mg/day doses was found to increase prolactin levels relative to placebo in the overall population. Treatment with olanzapine was found, by Leucht et al., to increase prolactin relative to placebo, which is consistent with the current review. While the review by Leucht et al. was also in patients with schizophrenia, the scope was not limited to children. This difference may explain discrepancies between findings.

Differences between male and female patients with respect to the prolactin elevation response, particularly the more pronounced responses among female patients, have been described in the literature. A 1992 study of schizophrenia patients found that serum prolactin increased more markedly in female patients following the commencement of neuroleptic medications, despite the female patients receiving lower doses of treatment [35]. Interestingly, the individual patient variability in response was also higher in females than males. These findings supported an earlier study also conducted in patients with schizophrenia [36]. Similar trends were observed in the current review, with females having a higher prolactin elevation response compared to males when treated with risperidone and quetiapine. However, an opposite trend was observed for patients treated with paliperidone 3–6 mg/day or 6–12 mg/day.

Typically, reference ranges for biochemical markers are established to inform physicians on levels at which these markers may pose a risk to patient safety. While the endocrinology literature documents the effect of prolactin on the body, no acceptable ranges have been established for serum prolactin levels in the pediatric population. A 1973 study of 19 normal children aged 2–12 years and 54 adolescent children aged 13–16 years estimated mean prolactin levels in both male and females to be approximately 5 ng/mL [37]. However, patient characteristics were poorly described, so generalizations must be made with caution. A study by Cook et al. proposed reference ranges (2.5 to 97.5 percentiles) for pediatric prolactin based on a hospital sample [38]. Based on this patient sample, the normal ranges for males and females aged 13 to 15 years of age were defined as 1.6–16.6 ng/mL and 3.0–14.4 ng/mL, respectively. However, the level of elevation in prolactin levels that warrants concern for adverse events in children has yet to be defined. According to some clinical investigators, however, prolactin levels between 18 ng/mL and 30 ng/mL are rarely associated with adverse events related to the hypothalamic-pituitary-gonadal axis, though prolonged elevations nearing 100 ng/mL necessitate clinical investigation [13]. It is, therefore, difficult to determine what prolactin levels substantially increase the risk of prolactin-related adverse events in these patients [39]. Therefore, although some trends emerged in the analysis of prolactin changes in patients treated with second-generation antipsychotics, the lack of causal association precludes conclusions being drawn between the use of any particular antipsychotic and prolactin-related adverse events.

This review is useful in that the process and design was well structured, with screening and data extraction conducted using a duplicate, consensus-based approach. Additionally, the body of evidence considered was broad, including both RCTs and observational studies. While observational studies may lack the methodological rigor of RCTs, they may still be considered an important source of information on treatment efficacy and safety. Despite this, this review presents some limitations. First, few trials were identified from the available literature and many were characterized by small sample sizes. Secondly, the observational studies suffered from significant heterogeneity in study populations, both in comparison to one another and to the RCT evidence base. Similarly, the patients represented in the evidence base were primarily Caucasians, which may limit the generalizability of the findings to the wider patient population. Follow-up time, prior treatment experience, and patient diagnoses also differed significantly in the observational studies. Thirdly, estimates of dispersion were large in each of the RCTs. Finally, while the mechanism is clear between prolactin and breast development/lactation, there is no conclusive research to confirm this association epidemiologically. In a similar vein, it was not possible, from the included studies, to directly associate changes in prolactin levels with patient adverse events. While this review was focused on pediatric patients with schizophrenia, in order to present outcomes for a well-defined clinical area, it must also be recognized that the potential for treatment-related prolactin elevation is not limited to this population. The risk and effects of prolactin elevation are an important clinical concern in other diagnostic areas and should be explored.

## Conclusion

In summary, while some trends in prolactin level changes from baseline emerged in the data, no definitive conclusions on the association between particular antipsychotic use and prolactin-related adverse events can be drawn. Nevertheless, clinician monitoring of prolactin-related adverse events is pertinent. Future trials examining the effects of antipsychotic medications for pediatric schizophrenia and schizophrenia spectrum disorder patients should emphasize reporting on safety outcomes, with studies of adequate duration to allow for the observation of these outcomes. Routine monitoring of serum prolactin levels in study participants is encouraged, as this may allow investigators to directly attribute adverse events to changes in prolactin.

## Additional files

Additional file 1:
**Figure S1.** Evaluation of RCTs with the Cochrane risk of bias assessment. (PDF 267 kb)
Additional file 2:
**Table S1.** Evaluation of included observational studies with the Newcastle-Ottawa Scale. (DOCX 25 kb)

## Abbreviations

BPRS: Brief psychiatric rating scale
CGAS: Children’s global assessment scale
CGI-S: Clinical global impressions scale
DSM-IV: Diagnostic and statistical manual of mental disorders, 4th edition
FGA: First-generation antipsychotic
ITT: Intent-to-treat
K-SADS-PL: Kiddie schedule for affective disorders and schizophrenia for school-age children, present and lifetime version
NOS: Newcastle-Ottawa scale
NR: Not reported
PANSS: Positive and negative syndrome scale
RCT: Randomized controlled trial
SD: Standard deviation
SGA: Second-generation antipsychotic.
