# Supplementary material for: Prolactin-related adverse events and change in prolactin levels in pediatric patients given antipsychotics for schizophrenia and schizophrenia spectrum disorders: A systematic review
Source: BMC Pediatr. 2016 Nov 9;16:181. doi: 10.1186/s12887-016-0710-y (PMC5101725; doi:10.1186/s12887-016-0710-y)
Supplement: Additional file 1: — Figure S1. Evaluation of RCTs with the Cochrane risk of bias assessment. (PDF 267 kb) [file 12887_2016_710_MOESM1_ESM.pdf]

	Random sequence generation (selection bias)	Allocation concealment (selection bias)	Blinding of participants and personnel (performance bias)	Blinding of outcome assessment (detection bias)	Incomplete outcome data (attrition bias)	Selective reporting (reporting bias)	Other bias
Findling et al 2008	?	?	+	?	−	+	−
Findling et al 2012	?	+	+	?	+	+	?
Haas et al 2009a	?	?	+	?	+	+	−
Haas et al 2009b	+	?	+	?	+	+	?
Kryzhanovskaya et al 2009	+	?	+	?	+	+	−
Singh et al 2011	+	+	+	?	+	+	?
