# Supplementary material for: Prolactin-related adverse events and change in prolactin levels in pediatric patients given antipsychotics for schizophrenia and schizophrenia spectrum disorders: A systematic review
Source: BMC Pediatr. 2016 Nov 9;16:181. doi: 10.1186/s12887-016-0710-y (PMC5101725; doi:10.1186/s12887-016-0710-y)
Supplement: Additional file 2: — Table S1. Evaluation of included observational studies with the Newcastle-Ottawa Scale. (DOCX 25 kb) [file 12887_2016_710_MOESM2_ESM.docx]

**Table A1: Evaluation of included observational studies with the Newcastle-Ottawa Scale**

| **Study** | **Duval et al 2008 [30]** | **Kumra et al 2008 [31]** | **Pandina et al 2012 [32]** | **Ruan et al 2010 [33]** | **Shimmelmann et al 2007 [34]** |
| --- | --- | --- | --- | --- | --- |
| **Representativeness of the exposed cohort** | * | * | * | * | * |
| **Selection of the non-exposed cohort** |  | * |  |  |  |
| **Ascertainment of exposure** | * | * | * | * | * |
| **Demonstration that outcome of interest was not present at start of study** | * | * | * | * | * |
| **Comparability of cohorts on the basis of the design or analysis** |  |  |  |  |  |
| **Assessment of outcome** | * | * | * | * | * |
| **Was follow-up long enough for outcomes to occur** | * | * | * | * | * |
| **Adequacy of follow-up of cohorts** | * | * | * | * | * |
| **Number of stars** | 6 | 7 | 6 | 6 | 6 |
